# Postpartum Dyspareunia Following Continuous Versus Interrupted Perineal Repair: A Systematic Review and Meta-Analysis

**DOI:** 10.7759/cureus.29070

**Published:** 2022-09-12

**Authors:** Emma M Schnittka, Nick W Lanpher, Praful Patel

**Affiliations:** 1 Medicine, Alabama College of Osteopathic Medicine, Dothan, USA; 2 Obstetrics and Gynecology, Alabama College of Osteopathic Medicine, Dothan, USA

**Keywords:** interrupted suture, continuous suture, episiotomy, perineal tear, dyspareunia

## Abstract

Research evaluating optimal repair techniques for the reduction of postpartum dyspareunia following obstetric laceration is severely limited. Prevailing guidelines from the American College of Obstetricians and Gynecologists (ACOG) are reliant on data from just nine clinical trials conducted from 1980 to 2012. While the literature on this topic is still limited today, this review aims to synthesize data from past and present studies to ensure that standing clinical recommendations are supported by current literature.

A review was conducted per Preferred Reporting Items for Systematic Reviews and Meta-Analyses (PRISMA) 2020 guidelines. Medical Literature Analysis and Retrieval System Online (MEDLINE), Cochrane Library, and Google Scholar were searched. Included articles (1) compared continuous with interrupted repair techniques for subjects with episiotomies and/or second-degree tears, (2) were available in full length, and (3) reported dyspareunia as an outcome variable. Excluded articles were those (1) inclusive of first-, third-, or fourth-degree tears; (2) comparing suture material rather than technique; and (3) not available in English. A meta-analysis was conducted for both acute dyspareunia (<3 months) and chronic dyspareunia (>3 months) utilizing Meta-Essentials Microsoft Excel (Microsoft Corp., Redmond, WA) workbook. Bias was evaluated via Egger regression and Begg and Mazumdar rank correlation tests.

Twelve articles met inclusion and exclusion guidelines, seven for acute dyspareunia and eight for chronic dyspareunia. All publications were randomized controlled trials and were inclusive of a total of 4,081 patients. Risk ratios (RRs) and 95% confidence intervals (CIs) were calculated using a random effect model. Analysis revealed no statistically significant difference between continuous and interrupted suture groups for acute dyspareunia (RR: 0.98; 95% CI: 0.89-1.08) or chronic dyspareunia (RR: 0.96; 95% CI: 0.83-1.12). Egger regression test (p-value=0.534) and Begg and Mazumdar rank correlation test (p-value=0.570) indicated minimal publication bias. Compiled data does not indicate a preferential suture technique for the reduction of postpartum dyspareunia. These findings are congruent with the ACOG guidelines; therefore, there is no supporting evidence for ACOG's recommendation of continuous suturing to be overturned.

## Introduction and background

Perineal trauma is a prevalent complication of standard vaginal birth, affecting 53%-75% of patients worldwide [1,2]. Of those lacerations obtained, first- and second-degree tears are the most common [1,3]. A first-degree tear involves the vaginal mucosa and perineal skin, while second-degree tears extend into the superficial and/or deep perineal musculature [1]. Likewise, episiotomy, the surgical enlargement of the vagina to aid in second-stage labor, occurs frequently. This procedure is most commonly indicated for shoulder dystocia, suspected fetal distress, and fetal malposition [4]. Though discontinued as a standard practice in 2006, 12% of US births still involve episiotomy [5]. Optimal suture techniques for the repair of these lacerations have long been studied. While much investigation has focused on reducing perineal pain and improving wound healing, research regarding postpartum dyspareunia, painful sexual intercourse, is limited [6].

Of the published studies analyzing perineal repair and dyspareunia, the meta-analysis conducted by Kettle et al. in 2012 is the most comprehensive [6]. This meta-analysis serves as the cornerstone for current guidelines implemented by the American College of Obstetricians and Gynecologists (ACOG) for the management of obstetric lacerations. Last updated in "Practice Bulletin 198" circa 2018, current recommendations favor continuous suturing with synthetic, absorbable material for repair of second-degree lacerations. Such interventions are associated with reduced pain for up to 10 days postpartum and fewer complications. These guidelines do not recognize significant differences in dyspareunia between the continuous and interrupted groups; however, only nine clinical trials met the inclusion criteria for Kettle et al.'s analysis at the time of publication [7]. This review and meta-analysis incorporates updated literature published within the 10 years since Kettle et al.'s original research. In doing so, we aim to ensure ACOG's prevailing guidelines for the repair of obstetric lacerations regarding postpartum dyspareunia are supported by current evidence.

## Review

Methods

Search Strategy

The Preferred Reporting Items for Systematic Reviews and Meta-Analyses (PRISMA) 2020 checklist guided our review [8]. Two databases were searched by authors ES and NL from inception to June 10, 2022. First, Medical Literature Analysis and Retrieval System Online (MEDLINE) via PubMed was accessed at pubmed.ncbi.nlm.nih.gov. Our search utilized a combination of Medical Subject Headings (MeSH) including (continuous versus interrupted) (Title and Abstract) OR (perineal tear) (Title and Abstract) OR (episiotomy) (Title and Abstract) +/- AND (dyspareunia). Filters were not used to limit results by publication date or study type.

**Figure 1:**
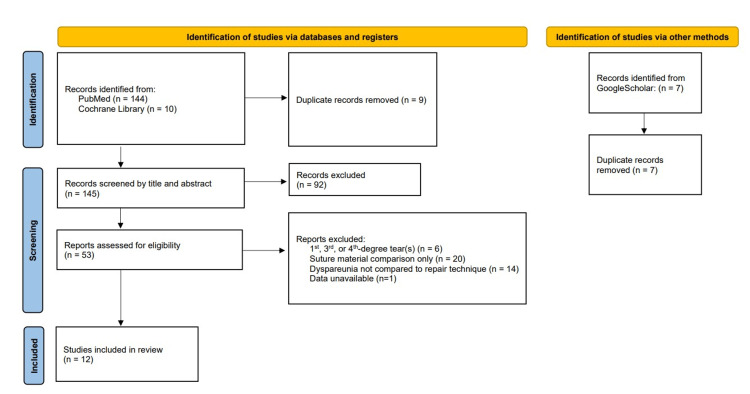
PRISMA 2020 flow diagram for new systematic reviews, which included searches of databases, registers, and other sources PRISMA: Preferred Reporting Items for Systematic Reviews and Meta-Analyses From: Page MJ, McKenzie JE, Bossuyt PM, Boutron I, Hoffmann TC, Mulrow CD, et al.: The PRISMA 2020 statement: an updated guideline for reporting systematic reviews. BMJ. 2021, 372:n71. 10.1136/bmj.n71. For more information, visit http://www.prisma-statement.org/

Cochrane Library via Wiley at cochranelibrary.com was also searched. A free-text search was conducted using relevant search terms including "perineal tear," "continuous suture," "interrupted suture," "episiotomy," and "dyspareunia." Additionally, a population, intervention, control, outcome (PICO) search was conducted using MeSH. Population was limited to patients with second-degree perineal tears or episiotomy. "Continuous suture technique" was selected as the intervention and "interrupted suture technique" as the control. "Dyspareunia" was selected as the appointed outcome. To account for unpublished or ongoing research, Google Scholar was searched using the same free-text terms as in our Cochrane Library search. A flowchart of this process is detailed in Figure 1.

Eligibility Criteria

Our initial search yielded 161 articles. Duplicates were removed, and remaining texts were filtered by title and abstract. All remaining literature was then reviewed in full and filtered by inclusion and exclusion criteria (Figure 1). Inclusion criteria were composed of the following: (1) compared continuous with interrupted suture techniques, (2) evaluated subjects with episiotomy and/or second-degree perineal tear, and (3) provided data for dyspareunia outcomes. Excluded studies were composed of the following: (1) evaluated first-, third-, and/or fourth-degree perineal tears; (2) compared suture material rather than suture technique; and (3) were not available in English.

Data Extraction

Twelve articles met selection criteria and were reviewed in full by authors independently. Nine of these articles [9-17] were included in Kettle et al.'s 2012 meta-analysis [6], while three were published in the following years [18-20]. Relevant data was recorded in Table 1.

**Table 1:** Summary of reviewed literature CG: continuous suture group; IG: interrupted suture group; VAS: visual analog scale

	Country of origin	Included in Kettle et al.'s 2012 analysis	Conclusion(s)	Notes
Almeida (2008) [9]	Brazil	Yes	No significant difference in dyspareunia; CG with reduced perineal pain four days postpartum	Dyspareunia data collected from CG at 49 days +/- 15.7 days and IG at 45.8 d +/-15.1, approximated at seven weeks postpartum
Morano (2006) [10]	Italy	Yes	No significant difference in dyspareunia; CG with reduced acute pain and analgesia use	
Valenzuela (2009) [11]	Spain	Yes	No significant difference in dyspareunia; CG with reduced suture time and amount of suture material used	
Kettle (2002) [12]	The United Kingdom	Yes	No significant difference in dyspareunia; CG with reduced perineal pain at 10 days postpartum	
Perveen (2009) [13]	Pakistan	Yes	No significant difference in dyspareunia; IG associated with increased number of suture packets used	
Mahomed (1989) [14]	England	Yes	CG with higher overall dyspareunia rate; IG subjects had increased need for removal of suture material	Potential bias due to variation in suture material, layers repaired, and injury type (episiotomy, tear, or both); authors note some non-compliance to allocated suture technique by midwives (values not given)
Isager-Sally (1986) [15]	Denmark	Yes	No significant difference in long-term dyspareunia; CG with reduced acute discomfort, incontinence, and dyspareunia and improved cosmetic results	Authors note that females experiencing dyspareunia were offered pelvic examination, which deduced "25% (of complaints) related directly to the episiotomy scar while in 75%, there were unrelated reasons"
Detlefsen (1980) [16]	Denmark	Yes	CG experienced reduced acute dyspareunia, with no significant difference in chronic dyspareunia; CG recommended due to reduced edema, subjective discomfort, and need for postpartum analgesia	
Croce (1997) [17]	Italy	Yes	No significant difference in dyspareunia; improved aesthetic in CG at four days and three months postpartum	
Kindberg (2008) [18]	Denmark	No	No significant difference in dyspareunia; CG with reduced suture time, amount of suture used, and cost	Low compliance to allocated suture technique by midwives (77% CG and 80% IG); potential bias due to the use of both rapidly absorbable and standard polyglactin 910
Kokanalı (2011) [19]	Turkey	No	No significant difference in dyspareunia; CG with reduced suturing time, suture material used, and perineal pain at one day postpartum	Pain rated using VAS scores, value of four or higher considered moderate/severe pain [17]; raw data not given, shown data calculated from z-scores of VAS ratings
Aydın Besen (2020) [20]	Turkey	No	No significant difference in dyspareunia; CG with reduced suture time, suture material used, perineal pain, and analgesia used; CG with improved wound healing	

Meta-Analysis

Two calculations were made using raw data from Tables 2, 3. The first meta-analysis included data regarding "acute" dyspareunia, defined as dyspareunia within three months of delivery. The second meta-analysis evaluated "chronic" dyspareunia, defined as dyspareunia at or beyond three months postpartum. Calculations were made using Meta-Essentials Microsoft Excel (Microsoft® Corp., Redmond, WA) workbooks [21,22]. Workbook 2, "Differences between independent groups - binary data," was selected based on the formatting of the raw data collected.

**Table 2:** Data summary: admission or denial of acute dyspareunia at listed intervals CG: continuous suture group; IG: interrupted suture group *Kindberg (2008) first intercourse timeframe not specified [8] **Valenzuela (2009) first intercourse: average of 49 days in CG and 45 days in IG [11]

	Suture	CG	IG	Admits, CG	Denies, CG	Admits, IG	Denies, IG
Almeida (2008) [9]	Polyglactin 910, rapidly absorbable	12	11	7 weeks: 5	7 weeks: 7	7 weeks: 5	7 weeks: 6
Valenzuela (2009) [11]	Polyglactin 910, rapidly absorbable	198	186	First intercourse**: 109	First intercourse: 89	First intercourse: 110	First intercourse: 76
Perveen (2009) [13]	Catgut	50	50	6 weeks: 3	6 weeks: 47	6 weeks: 2	6 weeks: 48
Perveen (2009) [13]	Polyglactin 910, standard	50	50	6 weeks: 3	6 weeks: 47	6 weeks: 3	6 weeks: 47
Croce (1997) [17]	Catgut	96	99	1 month: 32	1 month: 64	1 month: 27	1 month: 72
Detlefsen (1980) [16]	Polyglycolic acid	63	50	2 months: 48	2 months: 15	2 months: 45	2 months: 5
Kindberg (2008) [18]	Polyglactin 910, rapidly absorbable or standard	198	197	First intercourse*: 124	First intercourse: 74	First intercourse: 111	First intercourse: 86
Kokanalı (2011) [19]	Polyglactin 910, rapidly absorbable	10	9	6 weeks: 9	6 weeks: 1	6 weeks: 7	6 weeks: 2
Kokanalı (2011) [19]	Polyglycolide-co-caprolactone	9	11	6 weeks: 8	6 weeks: 1	6 weeks: 10	6 weeks: 1

**Table 3:** Data summary: admission or denial of chronic dyspareunia at listed intervals CG: continuous suture group; IG: interrupted suture group

	Suture	CG	IG	Admits, CG	Denies, CG	Admits, IG	Denies, IG
Morano (2006) [10]	Polyglactin 910, rapidly absorbable	87	78	3 months: 18	3 months: 69	3 months: 18	3 months: 60
Kettle (2002) [12]	Polyglactin 910, standard	298	290	3 months: 47	3 months: 251	3 months: 48	3 months: 242
Kettle (2002) [12]	Polyglactin 910, rapidly absorbable	283	303	3 months: 51	3 months: 232	3 months: 54	3 months: 249
Mahomed (1989) [14]	Polyglycolic acid, catgut, or silk	424	401	3 months: 116	3 months: 308	3 months: 94	3 months: 307
Isager-Sally (1986) [15]	Polyglycolic acid	265	250	3 months: 45	3 months: 220	3 months: 58	3 months: 192
Detlefsen (1980) [16]	Polyglycolic acid	63	50	6 months: 28	6 months: 35	6 months: 19	6 months: 31
Croce (1997) [17]	Catgut	96	99	3 months: 24	3 months: 72	3 months: 25	3 months: 74
Kindberg (2008) [18]	Polyglactin 910, rapidly absorbable or standard	198	197	6 months: 47	6 months: 151	6 months: 58	6 months: 139
Aydın Besen (2020) [20]	Unspecified	26	27	3 months: 6	3 months: 20	3 months: 11	3 months: 16

Evaluation of Bias 

All data (both acute and chronic) was combined in a single meta-analysis for evaluation of publication bias. A p-value of <0.05 was considered indicative of bias based on Egger regression test and Begg and Mazumdar rank correlation test calculations.

Results

Study Characteristics

All included studies were randomized controlled trials. Studies spanned eight different countries and were published from 1980 to 2020. These studies were inclusive of 4,081 patients, 2,069 with continuous intervention and 2,012 for interrupted intervention. Of these studies, seven relayed data for acute dyspareunia and eight for chronic dyspareunia. Only Kettle et al. (2002) evaluated data beyond six months [12]. This data was excluded to prevent skew.

Just two studies found statistically significant differences in dyspareunia when comparing continuous and interrupted sutures [14,16]. Every study favored continuous suturing for benefits unrelated to dyspareunia. Suture material varied across reviewed literature. Seven studies utilized only one type of suture material: Vicryl [9-11,18], Dexon [16], catgut [17], or unspecified [20]. The five remaining studies were comparative of dyspareunia relative to suture type [12-15,19]. No studies compared the same suture materials, and no significant differences in dyspareunia were noted between suture groups.

Acute and Chronic Dyspareunia

Data for acute dyspareunia (Table 2) was consistent with Kettle et al.'s 2012 analysis (RR: 0.98; 95% CI: 0.89-1.08). The evaluation of chronic dyspareunia (Table 3) yielded similar results (RR: 0.96; 95% CI: 0.83-1.12). These values indicate no significant difference in dyspareunia between continuous and interrupted suture recipients.

Publication Bias

Articles were assessed for quality by authors ES and NL independently. Quality evaluation considered study design (e.g., randomization and researcher blindness) and data interpretation (e.g., accurate conclusions drawn from p-values). Additionally, publication bias was quantitatively assessed via Egger regression and Begg and Mazumdar rank correlation tests, which revealed p-values of 0.534 and 0.570, respectively, indicating minimal bias. Other potential sources of bias are described in Table 1.

Discussion

Current ACOG guidelines recommend continuous suturing for the repair of episiotomies and second-degree perineal lacerations. This protocol is associated with reduced acute perineal pain and fewer complications, including the need for suture removal [7]. However, when these guidelines were issued in 2018, it was unclear if continuous suturing was associated with reduced postpartum dyspareunia. Our meta-analysis confirms previous data, revealing there is no significant difference in dyspareunia following continuous or interrupted perineal repair. These results endorse prevailing ACOG recommendations.

We consider the depth and scope of this review to be its greatest strength. Each article was evaluated by reviewers independently and thoroughly. Any potential bias or unexpected findings were explored. When reviewing Almeida (2008), we noted a substantial range in follow-up times, which was accounted for by categorizing acute data within a three-month timeframe rather than a singular quantitative value [9]. Additionally, Isager-Sally (1986) provided percentile data rather than raw data [15]. Such values were converted using z-scores to maintain homogeneity across studies; however, we are aware of the possible imprecision in this method. Potential sources of bias including low compliance and non-surgical causes of dyspareunia were also noted in Table 1 [14,15,18]. Furthermore, by conducting a literature search without filtering for date or study type, we are confident that all available work was included in our exploration.

The most significant weakness of this review is the limited pool of relevant studies. Despite the predominance of perineal laceration, our search yielded minimal results [1-3]. Of the twelve included studies, nine had been evaluated by Kettle et al. prior to 2012. In the decade following Kettle et al.'s research, just four additional articles were published on this topic. In addition to those detailed in this analysis [18-20], a clinical trial from Martínez-Galiano et al. in 2020 also compared dyspareunia between continuous and interrupted suture groups. The study concluded that continuous suturing reduced dyspareunia with statistical significance; however, this data was unavailable [23].

In addition to the lack of relevant publications, our systemic review and meta-analysis was complicated by variations in suture material, individual performance, and sample size. The reviewed literature was inclusive of five different types of suture; however, of the studies comparing suture material, no differences in dyspareunia were noted. Additionally, we acknowledge the variation in technical skill possessed by the individuals performing the suturing within and across studies. Differences in sample size were also noted. Group sizes ranged from nine to 303 patients. While p-values in our evaluation of publication bias were low, we acknowledge potential inaccuracy due to this discontinuity.

Overall, research regarding surgical repair of perineal lacerations and sexual dysfunction remains severely limited. Our work emphasizes the need for further investigation to determine optimal suturing techniques for reducing dyspareunia in the postpartum period.

## Conclusions

Our systematic review and meta-analysis suggests that there is no statistically significant difference in postpartum dyspareunia between patients with continuous and interrupted perineal repair. In synthesizing both contemporary and preceding data, our evaluation ensures that prevailing ACOG guidelines for the management of perineal tears are supported by evidence-based research.

This review has also emphasized the limited availability of research regarding the optimal reduction of dyspareunia in the postpartum period. We acknowledge that additional exploration is needed to verify our results, and we encourage the pursuit of future randomized controlled trials for the evaluation of suture techniques in the repair of obstetric lacerations.
